# Intermolecular Proton Transfer Enabled Reactive CO_2_ Capture by the Malononitrile Anion

**DOI:** 10.1021/acs.jpcb.4c04482

**Published:** 2024-10-02

**Authors:** Bo Li, Yuqing Fu, Zhenzhen Yang, Sheng Dai, De-en Jiang

**Affiliations:** † Department of Chemical and Biomolecular Engineering, Vanderbilt University, Nashville, Tennessee 37235, United States; ‡ Department of Chemistry, University of California, Riverside, California 92521, United States; § Chemical Sciences Division, Oak Ridge National Laboratory, Oak Ridge, Tennessee 37831, United States; ∥ Department of Chemistry, University of Tennessee, Knoxville, Tennessee 37996, United States

## Abstract

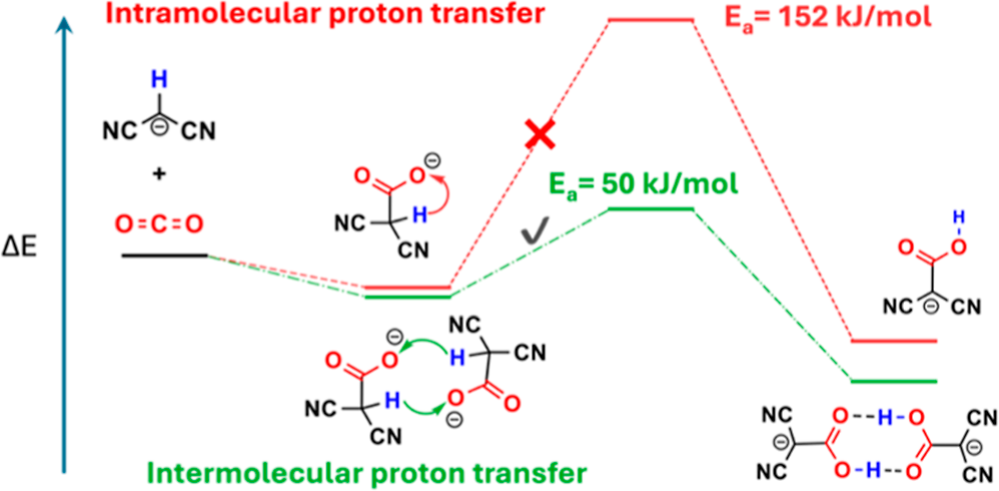

Task-specific ionic liquids (ILs) employing carbanions represent a new class of ILs for carbon capture. The deprotonated malononitrile carbanion, [CH(CN)_2_]^−^, has shown close to equimolar capacity for reactive CO_2_ capture. Although the formation of the [C(CN)_2_COOH]^−^ carboxylic acid was found to be the final product, how the hydrogen atom on the [CH(CN)_2_]^−^ carbanion transfers to the carboxylate group as a proton has not been fully understood. In this work, we employ density functional theory calculations with an implicit solvation model to investigate the proton transfer mechanisms in forming carboxylic acid from the reaction of the [CH(CN)_2_]^−^ carbanion with CO_2_. We find that the intramolecular proton-transfer pathway in [CH(CN)_2_COO]^−^ to form [C(CN)_2_COOH]^−^ is unlikely due to the high energy barrier of 152 kJ/mol. Instead, the intermolecular proton transfer pathway between two [CH(CN)_2_COO]^−^ anions is more feasible to form two molecules of [C(CN)_2_COOH]^−^, with a significantly lower activation energy of 50 kJ/mol. Moreover, the [C(CN)_2_COOH]^−^ dimer is further stabilized by the intermolecular hydrogen bonds of the two –COOH groups in the Z-configuration of the π-conjugated planar geometry. This insight of reactive CO_2_ capture enabled by intermolecular proton transfer will be useful in designing novel carbanions and ILs for carbon capture and conversion.

## 1 Introduction

Ionic liquids (ILs) are promising candidates for CO_2_ capture for their low vapor pressure, high thermal stability, and large chemical tunability.^1−3^ Task-specific ionic liquids (TSILs),^3−8^ including amino group-functionalized ILs (AILs),^9−14^ superbase-derived task-specific ionic liquids (STSILs),^15−21^ and ionic deep eutectic solvents,^22^ were developed for CO_2_ chemisorption to promote CO_2_ uptake capacity. A new type of carbanion-derived STSIL was recently developed by deprotonating the malononitrile molecule, CH_2_(CN)_2_, to achieve close to equimolar CO_2_ chemisorption (0.86 mol/mol) when paired with phosphonium cations such as P_66614_.^23^ Experimental and simulated ^1^H and ^13^C NMR and Fourier transform infrared spectra demonstrated the formation of carboxylic acid after the reaction of [P_66614_]^+^[CH(CN)_2_]^−^ with CO_2_.^23^ Quantum chemical calculations confirmed that the formation of [C(CN)_2_COOH]^−^ is thermodynamically more favorable than the carboxylate product, [CH(CN)_2_COO]^−^.^23^ However, the detailed mechanism of how the proton transfer takes place is not clear. Understanding such proton transfer processes can help the design and discovery of new anions and ILs for reactive CO_2_ capture because proton management has been shown to be important not only in conventional amine-based sorbents^24^ but also in ILs.^25,26^

**Scheme 1:**
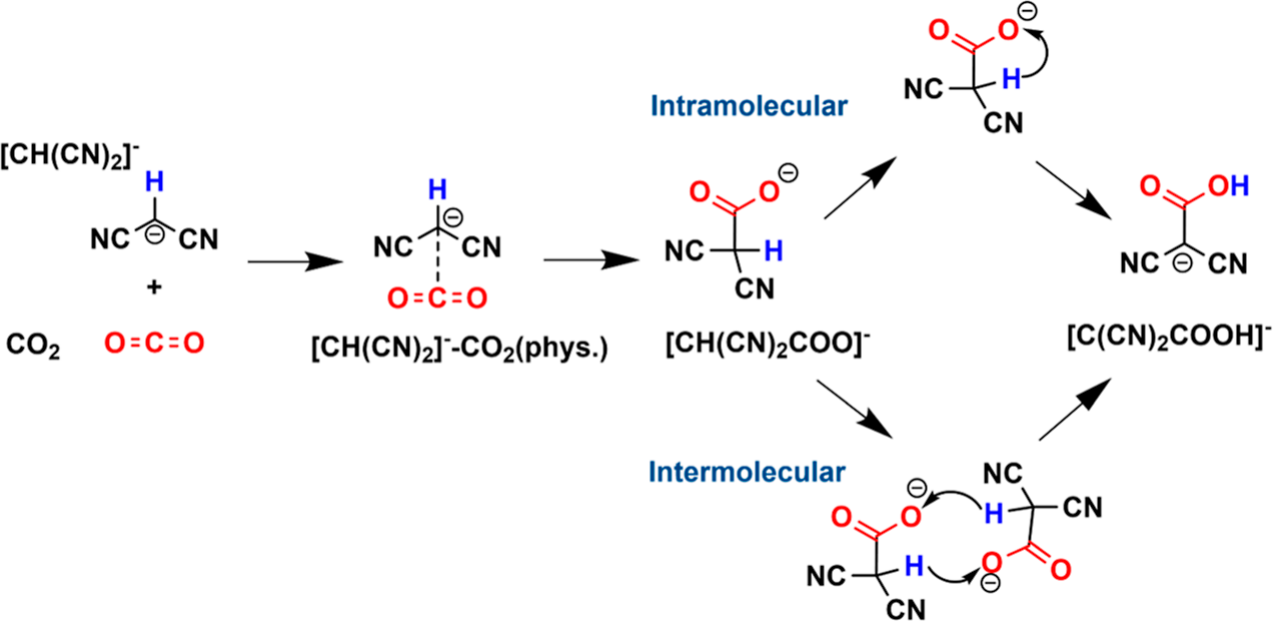
Proposed Reaction Pathways of the Deprotonated Malononitrile Carbanion, [CH(CN)_2_]^−^, with CO_2_ in Forming the Carboxylate Acid Product CO_2_(phys.) means a non-bonded physical interaction or physisorption state of CO_2_ with the [CH(CN)_2_]^−^ anion, prior to C–C bond formation leading to the carboxylate product, [CH(CN)_2_COO]^−^.

The Lewis acid–base chemistry between CO_2_ and [CH(CN)_2_]^−^ due to the nucleophilic attack of the [CH(CN)_2_]^−^ anion on the C atom of CO_2_ to form a carboxylate, [CH(CN)_2_COO]^−^, is facile with a computed free energy of activation of ∼47 kJ/mol.^27^ It has also been shown from quantum chemical calculations that the carboxylic acid product, [C(CN)_2_COOH]^−^, is about 25 kJ/mol more stable in energy than the carboxylate product.^23,27^ From [CH(CN)_2_COO]^−^ to [C(CN)_2_COOH]^−^, there are two likely routes for proton transfer from –CH to –COO^–^, as shown in Scheme 1: (i) intramolecular proton transfer to a carboxylate O atom in the same [CH(CN)_2_COO]^−^ anion and (ii) intermolecular proton transfers between two [CH(CN)_2_COO]^−^ anions. Intermolecular proton transfer mechanisms in reactive CO_2_ capture by amino acid ILs have been explored by *ab initio* molecular dynamics simulations recently, where an amino proton transfers to a carboxylate moiety.^25,26^ But it is unclear how these happen in the carbanion-based ILs.

The goal of the present work is to elucidate the mechanism of the capture of CO_2_ by the [CH(CN)_2_]^−^ anion, especially the detailed energetics of the proton transfer pathways. This mechanistic understanding will be useful to design novel carbanions for TSILs. We use density functional theory (DFT) calculations with an implicit solvation model, which allows us to focus on the reactivity of the anion. Below, we first explain the method details.

## 2 Computational Methods

All the DFT calculations were performed using the Gaussian 16 software package^28^ at the B3LYP/TZVP level of theory with Grimme’s D3 dispersion correction.^29−31^ The convergence criteria of 10^–8^ on the root-mean-square density matrix and 10^–6^ hartree on the energy change were applied for the electronic structure calculations. Geometry optimization and transition state (TS) search were carried out with the convergence criteria of 4.5 × 10^–4^ hartree/Bohr on forces. The Berny algorithm was used to search for the transition states.^32^ The [CH(CN)_2_]^−^ anion is a small, flat molecule with only one stable configuration, so the initial geometry bias is less an issue due to the limited single-bond rotation degrees of freedom. When examining the reactions of [CH(CN)_2_]^−^ with CO_2_, we have explored the not-so-many different initial structures for geometry optimization and transition search and reported only the lowest-energy values. Normal mode analysis was further performed to ensure that local minima were found in geometry optimization without imaginary frequencies, and the TS showed only one imaginary frequency. Solvation effect was included by using the SMD solvation model with the parameterization for generic ionic liquid (SMD-GIL),^33,34^ which was derived to reproduce 344 experimental solvation free energies of neutral solutes and 431 water-to-IL transfer energies for 11 different ILs. Solvent descriptors used for SMD-GIL were the dielectric constant (ε = 11.50), the index of refraction (*n* = 1.43), the macroscopic surface tension (γ = 61.24 cal mol^–1^ Å^–2^), Abraham’s hydrogen bond acidity and basicity parameters (∑α_2_^H^ = 0.229 and ∑β_2_^H^ = 0.265), and carbon aromaticity (ϕ = 0.2142).^34^

## 3 Results and Discussion

As shown in Scheme 1, the carboxylate product, [CH(CN)_2_COO]^−^, is a key intermediate in reactive CO_2_ capture by [CH(CN)_2_]^−^ to form [C(CN)_2_COOH]^−^. Therefore, we start with the step leading to the formation of [CH(CN)_2_COO]^−^.

### 3.1 CO_2_ Interaction with the Malononitrile Carbanion: From Physisorption to Carboxylate Formation

**Figure 1:**
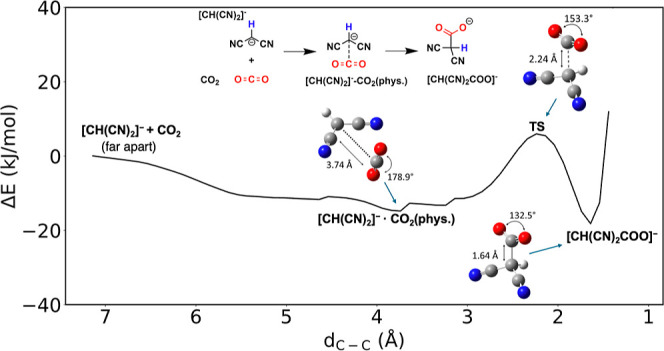
Potential energy surface of CO_2_ binding and reaction with the malononitrile carbanion, [CH(CN)_2_]^−^, with the C–C distance between the C atom of CO_2_ and the central C atom of the [CH(CN)_2_]^−^ anion as the reaction coordinate. The energy sum of separate [CH(CN)_2_]^−^ and CO_2_ molecules is set as zero. Color code: C, gray; H, white; O, red; N, blue.

We first examined the interaction between CO_2_ and the [CH(CN)_2_]^−^ carbanion by doing a relaxation scan with respect to the C–C distance between CO_2_ and the central C atom of [CH(CN)_2_]^−^. As shown in Figure 1, there are two local minima when the CO_2_ molecule approaches the [CH(CN)_2_]^−^ carbanion from afar (*d*_C–C_ > 7.0 Å). The first local minimum at *d*_C–C_ = 3.74 Å represents the physisorption state of CO_2_ with its linear geometry being maintained (∠_O–C–O_ = 178.9°).^35^ As *d*_C–C_ decreases further, the system overcomes an energy barrier of 20.8 kJ/mol to form a C–C bond between CO_2_ and [CH(CN)_2_]^−^: at the TS, *d*_C–C_ = 2.24 Å and ∠_O–C–O_ = 153.3° (being significantly bent). The relatively low activation energy confirms that the Lewis acid–base chemistry between CO_2_ and [CH(CN)_2_]^−^ to form a carboxylate is indeed facile. At the local minimum, the C–C bond is formed with *d*_C–C_ = 1.64 Å and ∠_O–C–O_ = 132.5°, corresponding to the formation of the carboxylate product, [CH(CN)_2_COO]^−^. The energy of [CH(CN)_2_COO]^−^ is −18.2 kJ/mol (with respect to the sum of separate CO_2_ and [CH(CN)_2_]^−^) and only 3.4 kJ/mol lower than the physisorption state, acting as the intermediate before the final chemisorption product of carboxylic acid.^23^ From [CH(CN)_2_]^−^ to [CH(CN)_2_COO]^−^, the central C atom changes from sp^2^ to sp^3^, as evidenced by the change of geometry from planar to nonplanar.

### 3.2 Intramolecular Proton Transfer Pathway for Carboxylic Acid Formation

**Figure 2:**
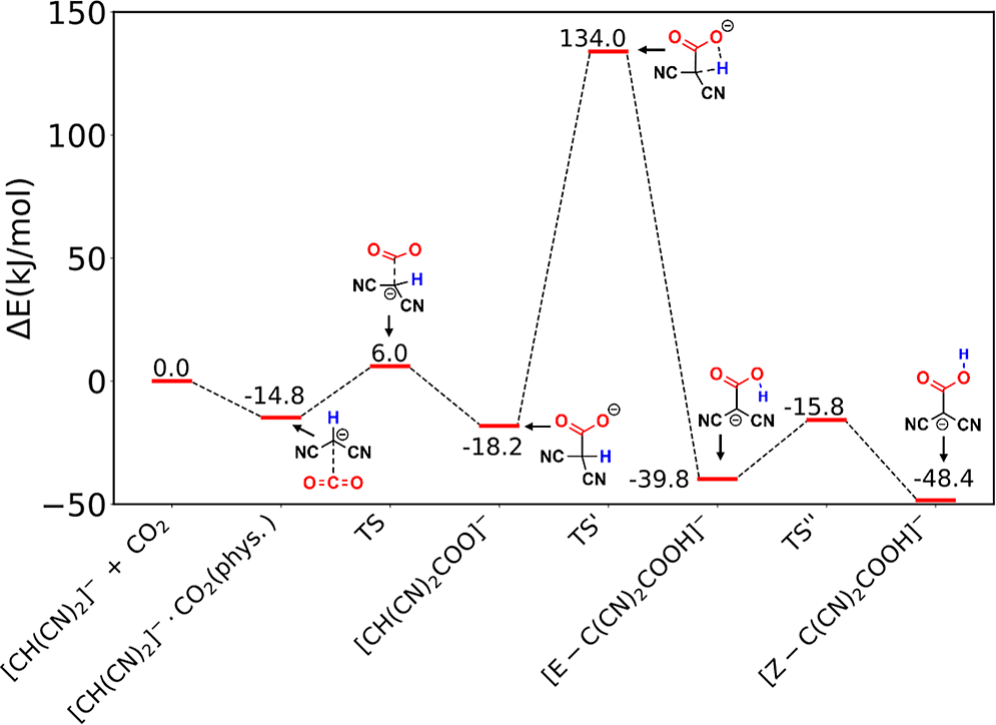
Energy diagram of CO_2_ binding and reaction with [CH(CN)_2_]^−^ to form [C(CN)_2_COOH]^−^ via the [CH(CN)_2_COO]^−^ intermediate and the intramolecular proton transfer pathway. The energy sum of the separate [CH(CN)_2_]^−^ and CO_2_ molecules is set as zero; the difference between the O–H and C–H distances of the transferring H is used as the reaction coordinate for the proton transfer step (TS′).

**Figure 3:**
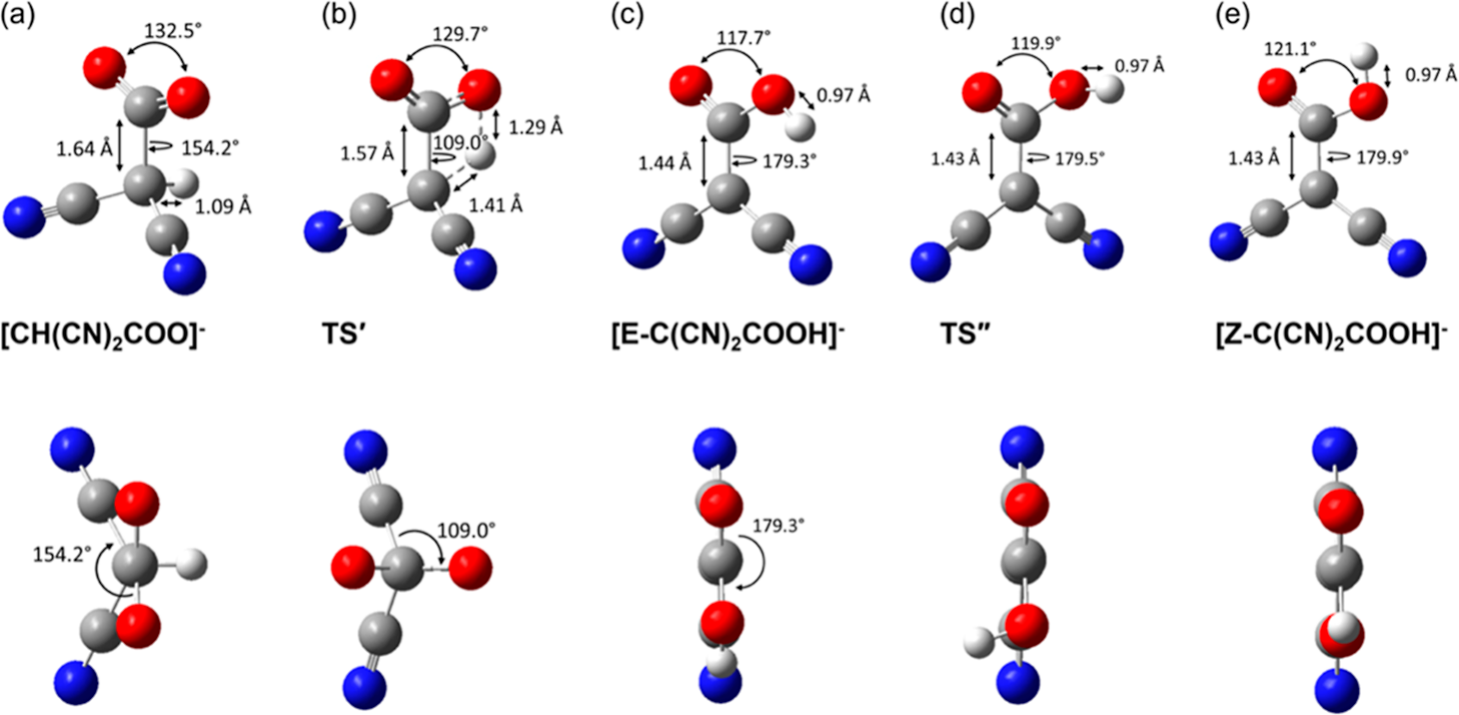
Optimized geometries along the path of CO_2_ reaction with [CH(CN)_2_]^−^ to form [C(CN)_2_COOH]^−^ via [CH(CN)_2_COO]^−^ and intramolecular proton transfer (top: perspective view; bottom: projection view along the C–C bond formed): (a) [CH(CN)_2_COO]^−^; (b) TS′; (c) [E-C(CN)_2_COOH]^−^; (d) TS″; and (e) [Z-C(CN)_2_COOH]^−^. See Figure 2 for the reaction path. Color code: C, gray; H, white; O, red; N, blue.

We next investigated the intramolecular proton transfer mechanism from [CH(CN)_2_COO]^−^ to the carboxylic acid product, [C(CN)_2_COOH]^−^. As shown in Scheme 1, the intramolecular proton transfer pathway involves the transfer of the hydrogen atom in [CH(CN)_2_COO]^−^ from the central C atom to one O atom on the COO^–^ moiety on the same [CH(CN)_2_COO]^−^ anion. Figure 2 shows the whole pathway from the physisorbed state to carboxylate formation then to carboxylic acid formation via intramolecular proton transfer, while Figure 3 shows the detailed geometries along the proton transfer process. We located the TS for the intramolecular proton transfer process (TS′), which has a four-membered ring structure and an energy barrier of 152.2 kJ/mol. From the [CH(CN)_2_COO]^−^ state to the TS′ state, the C–H bond increases from 1.09 to 1.41 Å in length and the O–H bond forms at 1.29 Å, while the dihedral φ_O–C–C–CN_ changes from 154.2 to 109° to bring H closer to a carboxylate of O (Figure 3b). After the proton transfer, the dihedral φ_O–C–C–CN_ rotates back and changes to 179.3°; in other words, the central C atom changes back to sp^2^ hybridization of the [CH(CN)_2_]^−^ anion to recover the π-conjugation and the [C(CN)_2_COOH]^−^ product is planar (Figure 3c). Due to the π-conjugation, the length of the C–C bond formed also shortens from 1.64 Å in [CH(CN)_2_COO]^−^ (Figure 3a) to 1.44 Å in [C(CN)_2_COOH]^−^ (Figure 3c).

Due to the re-established π-conjugation and the planar geometry of the [C(CN)_2_COOH]^−^ product, there are two different isomers along the C–OH bond in the CCOH plane: right after proton transfer, the [E-C(CN)_2_COOH]^−^ configuration is formed where the H atom is opposite the carbonyl O (Figure 3c); overcoming a small barrier of 24 kJ/mol (TS″ in Figures 2 and 3d) by rotating the O–H bond around the C–OH bond, the [E-C(CN)_2_COOH]^−^ isomer changes to the slightly more stable [Z-C(CN)_2_COOH]^−^ configuration where the H atom is on the same side with the carbonyl O with respect to the C–OH bond (Figure 3e). Another interesting trend of these chemical transformations is the change of the partial charge on the H atom: from [CH(CN)_2_]^−^ to [CH(CN)_2_COO]^−^ to [E-C(CN)_2_COOH]^−^ to [Z-C(CN)_2_COOH]^−^, the natural-bond-orbital charge on the H atom increases from 0.261 to 0.321 to 0.498 to 0.504 correspondingly. In other words, the H atom becomes more protic or Brønsted-acidic after the Lewis acid–base reaction between [CH(CN)_2_]^−^ and CO_2_ and the subsequent H transfer reaction and E-Z isomerization. Moreover, the more protic H atom becomes the stronger H-bond acceptor, which is key to the intermolecular proton transfer that we examine next.

### 3.3 Intermolecular Proton Transfer Pathway for Carboxylic Acid Formation

**Figure 4:**
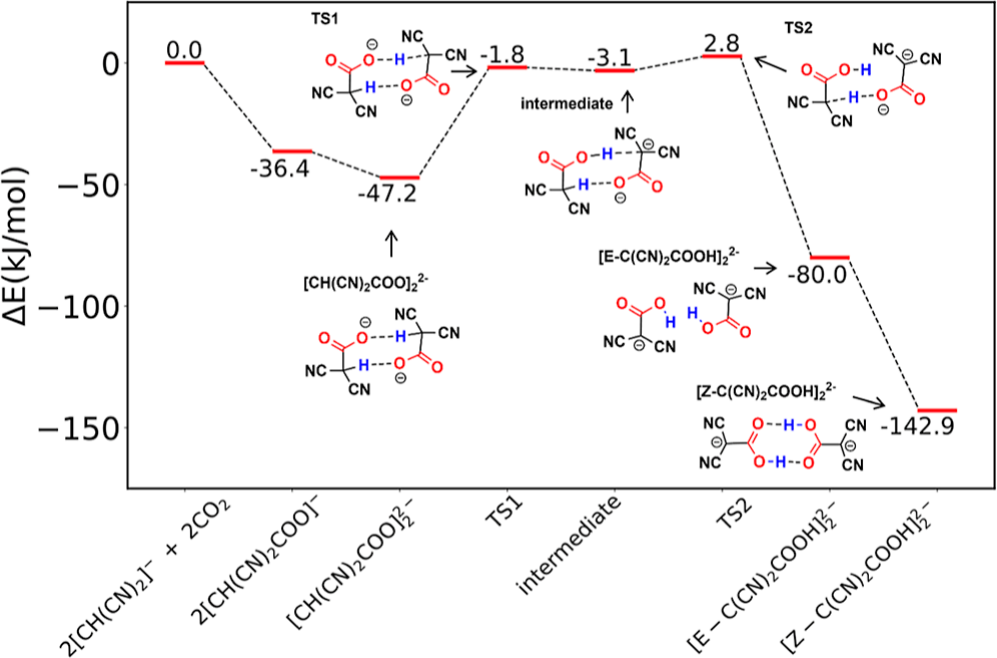
Energy profile of the intermolecular proton transfer between two carboxylate products from the reaction of the malononitrile anion with CO_2_ to form carboxylic acid. The energy sum of two separate carbanions and two separate CO_2_ molecules is set as zero.

**Figure 5:**
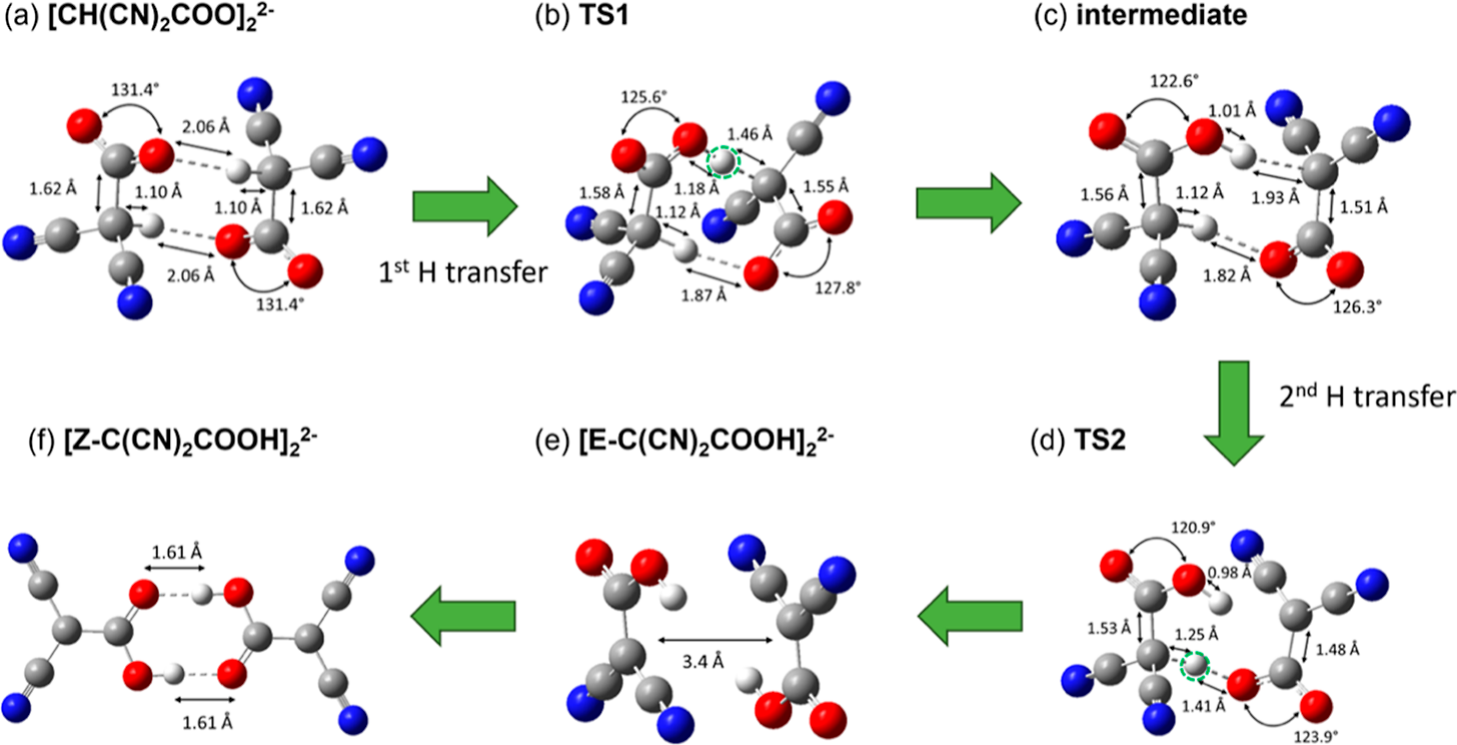
Optimized geometries along the reaction path of two CO_2_ molecules with two [CH(CN)_2_]^−^ anions to form [Z-C(CN)_2_COOH]_2_^2–^ via intermolecular proton transfers: (a) [CH(NC)_2_COO]_2_^2–^; (b) TS1; (c) intermediate between two proton transfers; (d) TS2; (e) [E-C(CN)_2_COOH]_2_^2–^; (f) [Z-C(CN)_2_COOH]_2_^2–^. See Figure 4 for the reaction path. Color code: C, gray; H, white; O, red; N, blue. Dashed lines denote hydrogen-bonds; the transferring H is highlighted with a dashed green circle in TS1 and TS2.

To investigate this pathway, we first optimized the energy and geometry of the dimer of the carboxylate product. Interestingly, we found that the carboxylate dimer ([CH(CN)_2_COO]_2_^2–^) is actually more stable than the sum of two isolated [CH(CN)_2_COO]^−^ by 10.8 kJ/mol (Figure 4). As can been seen from the structure of [CH(CN)_2_COO]_2_^2–^ (Figure 5a), the two intermolecular hydrogen bonds effectively distribute the two negative charges, leading to a more stable dianionic state; in addition, the structure is rather symmetric. From this state, we found that the two proton transfers take place in succession: the first transfer has an activation energy of 45.4 kJ/mol (TS1 in Figure 4) and, from [CH(CN)_2_COO]_2_^2–^ to TS1, the C–H bond of the transferring H increases from 1.10 to 1.46 Å while the O–H bond shortens from 2.06 to 1.18 Å (Figure 5b). After the first proton transfer is complete, the system transitions to a very shallow intermediate state consisting of the neutral [CH(CN)_2_COOH]^0^ and the [C(CN)_2_COO]^2–^ dianion, interacting with each other via two intermolecular hydrogen bonds (Figure 5c).

The second proton transfer starting with the shallow intermediate state has only a small barrier of 5.9 kJ/mol, and the energy of TS2 is only slightly higher than that of TS1 (Figure 4). At TS2 (Figure 5d) the C–H bond of [CH(CN)_2_COOH]^0^ is elongated to 1.25 Å, while the O–H bond is formed with one carboxylate O atom of [C(CN)_2_COO]^2–^ at 1.41 Å. After the transfer, the energy decreases by 82.8 kJ/mol and two [E-C(CN)_2_COOH]^−^ products are formed, aligned in parallel about 3.4 Å apart (Figure 5e). Of note, the [E-C(CN)_2_COOH]_2_^2–^ dimer (Figure 4; Δ*E* = −80.0 kJ/mol) is only 0.5 kJ/mol lower in energy than the sum of two isolated [E-C(CN)_2_COOH]^−^ (Figure 2; Δ*E* = −39.75 kJ/mol × 2 = −79.5 kJ/mol); in other words, the electrostatic repulsion between the two [E-C(CN)_2_COOH]^−^ anions is balanced off by their dispersion interaction in the [E-C(CN)_2_COOH]_2_^2–^ dimer. The two [E-C(CN)_2_COOH]^−^ anions can transition to the more stable Z-configuration, which can be further stabilized by intermolecular hydrogen bonds, as shown in Figure 5f: the total energy of [Z-C(CN)_2_COOH]_2_^2–^ is now down to −142.9 kJ/mol, that is, −71.4 kJ/mol per CO_2_ in terms of the reaction of [CH(CN)_2_]^−^ with CO_2_ to form [Z-C(CN)_2_COOH]^−^. This is in contrast to the −48.4 kJ/mol per CO_2_ driving force in the anion monomer formation (Figure 2).

### 3.4 Cation Influence and Entropy Contribution

**Figure 6:**
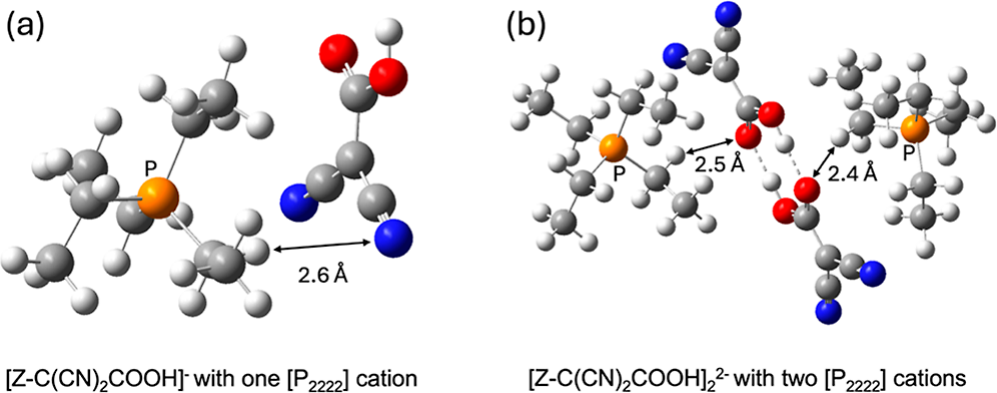
Optimized geometries of [Z-C(CN)_2_COOH]^−^ with the [P_2222_] cation presence: (a) from the reaction of [P_2222_][CH(CN)_2_] + CO_2_; (b) from the reaction of 2[P_2222_][CH(CN)_2_] + 2CO_2_. C, gray; H, white; O, red; N, blue; P, orange.

The present work focuses on the reaction of CO_2_ with the [CH(CN)_2_]^−^ anion. Although it is still challenging to simulate the condensed-phase reactions with explicit solvation, one could add a few explicit cations to test their influence on the CO_2_-anion reactions. We used tetraethylphosphonium ([P_2222_]^+^) as a model cation and recomputed the key reaction energies (Table 1) and product geometries (Figure 6) in the implicit solvation model. We found that the conclusion remains the same qualitatively: [Z-C(CN)_2_COOH]^−^ is still the most stable product for both the monomer and the dimer formations (Table 1). In addition, we found that the presence of cation stabilizes more the dimer product than the monomer product due to the multiple interactions between the methylene H atoms of [P_2222_]^+^ and the carbonyl O atoms of [Z-C(CN)_2_COOH]^−^ in the dimer (Figure 6b).

**Table 1:** Calculated Reaction Energies for Reactive CO_2_ Capture by the [CH(CN)_2_]^–^ Anion without and with the Explicit Tetraethylphosphonium ([P_2222_]^+^) Cation^a^

reactant	product	Δ*E* (kJ/mol)	
		without cation	with cation
[CH(CN)_2_]^−^ + CO_2_	[CH(CN)_2_COO]^−^	–18.2	–10.3
	[E-C(CN)_2_COOH]^−^	–39.8	–43.3
	[Z-C(CN)_2_COOH]^−^	–48.4	–52.1
2[CH(CN)_2_]^−^ + 2CO_2_	[CH(CN)_2_COO]_2_^2–^	–23.6	–30.6
	[E-C(CN)_2_COOH]_2_^2–^	–40.0	–53.5
	[Z-C(CN)_2_COOH]_2_^2–^	–71.4	–90.0

a All numbers in the table are based on per mole of CO_2_. The energy sum of separate carbanions and CO_2_ molecules is set as zero.

To consider the entropy contribution, we have calculated the enthalpies, entropies, and free energies at ambient conditions (*T* = 298.15 K and *P* = 1 bar), and the results are shown in Table 2. As can be seen, the Δ*H* values are similar to Δ*E*, confirming the energetic favorability for formation of the proton-transfer product in the Z-configuration and in the dimeric form. On the other hand, this enthalpic driving force is compensated or offset by the negative Δ*S* due to the loss of entropy from CO_2_ reacting with the anion and the dimer formation. As a result, the Δ*G* is rather neutral. If we take into account the presence of cations (Table 1), we expect that Δ*G* values would be slightly negative. On the other hand, the neutral Δ*G* values facilitate the desorption of CO_2_. Indeed, it has been shown experimentally that desorption of CO_2_ from the [P_66614_][CH(CN)_2_] IL can be achieved by bubbling N_2_ through the IL at 333 K for 30 min.^23^

**Table 2:** Energy (Δ*E*), Enthalpy (Δ*H*), Entropy (Δ*S*), and Free Energy (Δ*G*) Changes of CO_2_ Reaction with [CH(CN)_2_]^−^ at 298 K and 1 bar^a^

reactant	product	Δ*E* (kJ/mol)	Δ*H* (kJ/mol)	Δ*S* (J/mol/K)	Δ*G* (kJ/mol)
[CH(CN)_2_]^−^ + CO_2_	[CH(CN)_2_COO]^−^	–18.2	–12.7	–149.1	31.8
	[E-C(CN)_2_COOH]^−^	–39.8	–32.6	–153.8	13.3
	[Z-C(CN)_2_COOH]^−^	–48.4	–40.8	–156.0	5.7
2[CH(CN)_2_]^−^ + 2CO_2_	[CH(CN)_2_COO]_2_^2–^	–23.6	–15.3	–212.8	48.1
	[E-C(CN)_2_COOH]_2_^2–^	–40.0	–29.9	–233.6	39.8
	[Z-C(CN)_2_COOH]_2_^2–^	–71.4	–61.9	–225.2	5.2

a All numbers in the table are based on per mole of CO_2_ without an explicit cation. The energy sum of separate carbanions and CO_2_ molecules is set as zero.

### 3.5 Implications

Our results above not only confirm the intermolecular proton transfer being the preferred pathway for formation of the carboxylic product but also show the more favorable thermodynamic driving force when two [CH(CN)_2_]^−^ work together to capture two molecules of CO_2_. The intermolecular proton transfer pathway via hydrogen bonds was also found to be kinetically more facile in reactive CO_2_ capture by deprotonated amino acid,^25,26^ with a reaction stoichiometry of anion/CO_2_ = 2:1, forming a carbamate dianion and a neutral amino acid. So the intermolecular hydrogen bonds facilitate both proton transfer and the formation of a –COOH group. These intermolecular hydrogen bonds were also found to be important in nonaqueous amine systems to achieve equimolar CO_2_ capture with carbamic acid formation^24^ and in water-lean amine solvents that form carbamate anhydride with CO_2_ via a tetrameric self-assembly.^36^ Hence, leveraging the intermolecular hydrogen bonds is an important strategy to promote the capture of reactive CO_2_ by novel anions. Of note, the [P_66614_][CH(CN)_2_] IL has a relatively high viscosity of 1235 cP at room temperature.^23^ Although the viscosity of the IL after CO_2_ capture has not been measured, we expect that the enhanced intermolecular hydrogen bonding from the proton-transfer product might further increase the viscosity. Therefore, it would be worthwhile for future work to take viscosity into account in designing and tuning new carbanion-based ILs for reactive CO_2_ capture.

Another important question is how to experimentally verify the predicted intermolecular proton transfer pathway. The most straightforward approach is to measure the reaction rate of the malononitrile anion with CO_2_ at different temperatures and derive the activation energy, which can then be compared with our predicted values. Another approach is to measure the kinetic-isotope effect by using a deuterated malononitrile anion. The measured *k*_D_/*k*_H_ can then be compared with the simulated values for the intermolecular vs intramolecular pathways based on the computed reaction profiles. We hope that future experimental work will be undertaken to validate and confirm the theoretical predictions presented in this study.

## 4 Conclusions

We performed DFT calculations with the SMD solvation model for generic ILs to examine the reaction mechanism of [CH(CN)_2_]^−^ with CO_2_ to form the [C(CN)_2_COOH]^−^ product. We found that the formation of the carboxylate product, [CH(CN)_2_COO]^−^, is kinetically facile from the physisorption state of CO_2_ with [CH(CN)_2_]^−^. The intramolecular proton transfer from the central carbon in [CH(CN)_2_COO]^−^ to the COO^–^ moiety was found to have a high activation energy of 152 kJ/mol. In contrast, the intermolecular H transfer between two [CH(CN)_2_COO]^−^ anions has a significantly lower energy barrier of 50 kJ/mol, facilitated by the intermolecular hydrogen bonds between the two H atoms and the two COO^–^ moieties. Moreover, the intermolecular hydrogen bonds between the two –COOH groups via the Z-configuration of the planar geometry further stabilize the carbanion dimer, [Z-C(CN)_2_COOH]_2_^2–^. In sum, there are three main driving forces in the reactive CO_2_ capture by the [CH(CN)_2_]^−^ carbanion to form the carboxylic acid product: (i) the tendency for the carbanion to maintain the π-conjugation; (ii) the intermolecular hydrogen bonding to bring H in [CH(CN)_2_COO]^−^ close to the carboxylate group of another [CH(CN)_2_COO]^−^ and stabilize the anion dimer to facilitate proton transfer; (iii) further stabilization of the [C(CN)_2_COOH]^−^ dimer in the Z-configuration via hydrogen bonds between the two –COOH groups. The present work has quantitatively mapped out the energetics of proton transfer pathways in the CO_2_ capture chemistry by a carbanion via computational chemistry. Our findings will be useful in designing novel carbanion-based ILs for reactive CO_2_ capture.

## Supporting Information Available

Gaussian input files for key structures (PDF)
