# Supplementary material for: Intermolecular Proton Transfer Enabled Reactive CO2 Capture by the Malononitrile Anion
Source: J Phys Chem B. 2024 Oct 2;128(41):10207–13. doi: 10.1021/acs.jpcb.4c04482 (PMC11492316; doi:10.1021/acs.jpcb.4c04482)
Supplement: Supplementary file 1 — jp4c04482_si_001.pdf [file jp4c04482_si_001.pdf]

Supporting Information

Intermolecular Proton Transfer Enabled Reactive CO_2 Capture by the Malononitrile Anion

Bo Li,[1,†] Yuqing Fu,[2,†] Zhenzhen Yang,[3] Sheng Dai,[3,4] De-en Jiang[1,*]

[1] Department of Chemical and Biomolecular Engineering, Vanderbilt University, Nashville, TN 37235, USA

[2] Department of Chemistry, University of California, Riverside, California 92521, United States

[3] Chemical Sciences Division, Oak Ridge National Laboratory, Oak Ridge, Tennessee 37831, United States

[4] Department of Chemistry, University of Tennessee, Knoxville, Tennessee 37996, United States

[†]Equal contribution

*Corresponding author: de-en.jiang@vanderbilt.edu

Gaussian inputs for the key structures

#P b3lyp/tzvp opt em=gd3bj freq nosymm scrf=(SMD,Solvent=Generic,Read)

$[CH(CN)_2]^-$

-1 1

N	1.01464	-0.10676	-0.25143
C	2.16372	0.05638	-0.26970
C	3.61531	0.24893	-0.25699
C	4.13021	0.79454	-1.51457
N	4.56631	1.22644	-2.49980
H	3.88445	0.92041	0.56632

Eps=11.50
EpsInf=2.0449
HbondAcidity=0.229
HbondBasicity=0.265
SurfaceTensionAtInterface=61.24
CarbonAromaticity=0.2142
ElectronegativeHalogenicity=0

#P b3lyp/tzvp opt em=gd3bj freq nosymm scrf=(SMD,Solvent=Generic,Read)

$[CH(CN)_2COO]^-$

-1 1

N	0.91892	0.02869	-0.66115
C	2.05440	0.17279	-0.52542
C	3.49500	0.34779	-0.47698
C	3.91739	0.92066	-1.74266
N	4.17573	1.33353	-2.78730
H	3.75992	1.02175	0.34226
O	3.62805	-1.81339	0.59264
C	4.28158	-1.05525	-0.12803
O	5.41847	-1.11001	-0.60289

Eps=11.50
EpsInf=2.0449
HbondAcidity=0.229
HbondBasicity=0.265
SurfaceTensionAtInterface=61.24
CarbonAromaticity=0.2142
ElectronegativeHalogenicity=0

#P b3lyp/tzvp opt=(ts,calcfc,noeigen,ModRedundant) em=gd3bj freq nosymm scrf=(SMD,Solvent=Generic,Read)

TS′

```
-1 1
N      0.88782    -0.00737    -0.53015
C      2.02727     0.15646    -0.52946
C      3.46785     0.33839    -0.52351
C      3.92854     0.91804    -1.77268
N      4.30693     1.36226    -2.76493
H      5.20091     0.17788    -0.11651
O      3.62220    -1.80513     0.58287
C      4.26653    -1.04278    -0.14524
O      5.41449    -1.09357    -0.59900

B 6 3
B 6 9
B 3 8
A 9 8 7

Eps=11.50
EpsInf=2.0449
HbondAcidity=0.229
HbondBasicity=0.265
SurfaceTensionAtInterface=61.24
CarbonAromaticity=0.2142
ElectronegativeHalogenicity=0
```

#P b3lyp/tzvp opt em=gd3bj freq nosymm scrf=(SMD,Solvent=Generic,Read)

[E-C(CN)$_2$COOH]$^-$

-1 1

H	4.55988	0.61179	1.93407
N	5.02533	-3.27058	5.05594
C	4.88330	-2.48993	4.22025
C	4.80273	-1.49099	3.16963
C	6.15489	-1.11952	2.78799
N	7.25020	-0.85958	2.54149
O	3.07865	-0.40767	4.46570
C	3.90033	-0.20340	3.56986
O	4.14244	0.79675	2.87523

Eps=11.50
EpsInf=2.0449
HbondAcidity=0.229
HbondBasicity=0.265
SurfaceTensionAtInterface=61.24
CarbonAromaticity=0.2142
ElectronegativeHalogenicity=0

#P b3lyp/tzvp opt em=gd3bj freq nosymm scrf=(SMD,Solvent=Generic,Read)

[Z-C(CN)$_2$COOH]$^-$

-1 1

H	3.61920	2.08090	2.95919
N	4.45947	-3.36451	4.22525
C	4.65041	-2.29277	3.82129
C	4.87912	-1.00367	3.32256
C	6.08188	-0.74009	2.65741
N	7.07530	-0.48806	2.10920
O	2.87648	-0.13027	4.22191
C	3.88105	0.01297	3.54183
O	4.08493	1.22996	2.95434

Eps=11.50
EpsInf=2.0449
HbondAcidity=0.229
HbondBasicity=0.265
SurfaceTensionAtInterface=61.24
CarbonAromaticity=0.2142
ElectronegativeHalogenicity=0

#P b3lyp/tzvp opt em=gd3bj freq nosymm scrf=(SMD,Solvent=Generic,Read)

$[CH(CN)_2]_2^{2-}$

-2 1

N	1.69326	-1.03063	0.32093
C	2.46013	-0.14506	0.35101
C	3.34046	0.90240	0.13787
C	3.45340	1.20481	-1.20873
N	3.41293	1.30555	-2.37575
H	3.87740	1.40253	0.92638
H	1.66579	-2.04685	2.90660
C	2.22565	-1.64022	3.73507
C	2.94959	-0.46726	3.60257
N	3.58912	0.51330	3.57228
C	2.28280	-2.27560	4.96396
N	2.38753	-2.74829	6.03045

Eps=11.50
EpsInf=2.0449
HbondAcidity=0.229
HbondBasicity=0.265
SurfaceTensionAtInterface=61.24
CarbonAromaticity=0.2142
ElectronegativeHalogenicity=0

#P b3lyp/tzvp opt em=gd3bj freq nosymm scrf=(SMD,Solvent=Generic,Read)

$[CH(CN)_2COO]_2^{2-}$

-2 1

N	1.52711	-0.42534	-0.01712
C	2.65959	-0.21126	-0.02514
C	4.07458	0.10302	-0.12341
C	4.24043	1.14892	-1.11617
N	4.29720	1.96606	-1.92702
H	4.40631	0.46760	0.86237
O	4.57352	-2.22381	0.18010
C	4.97160	-1.21669	-0.42624
O	5.93550	-1.02807	-1.17161
H	4.31859	-1.92369	2.28227
N	5.02533	-3.27058	5.05594
C	4.88330	-2.48993	4.22025
C	4.80273	-1.49099	3.16963
C	6.15489	-1.11952	2.78799
N	7.25020	-0.85958	2.54149
O	3.07865	-0.40767	4.46570
C	3.90033	-0.20340	3.56986
O	4.14244	0.79675	2.87523

Eps=11.50
EpsInf=2.0449
HbondAcidity=0.229
HbondBasicity=0.265
SurfaceTensionAtInterface=61.24
CarbonAromaticity=0.2142
ElectronegativeHalogenicity=0

#P b3lyp/tzvp opt=(ts,calcfc,noeigen,ModRedundant) em=gd3bj freq nosymm scrf=(SMD,Solvent=Generic,Read)

TS1

-2 1

N	2.04974	-2.10740	0.33444
C	2.80431	-1.24941	0.14682
C	3.73963	-0.19239	-0.03384
C	3.20564	0.93362	-0.72287
N	2.78761	1.87943	-1.24344
H	4.01701	0.29196	1.31106
O	5.57063	-1.70272	0.07497
C	5.17479	-0.62348	-0.44288
O	5.81356	0.15002	-1.17885
H	5.21579	-1.55619	1.93219
N	2.78843	-2.33789	3.77399
C	3.80045	-1.90976	3.43464
C	5.07425	-1.33944	3.01367
C	6.17590	-1.94854	3.74634
N	7.05663	-2.43399	4.30306
O	5.69541	0.73928	4.08347
C	5.05830	0.22658	3.18323
O	4.32031	0.84458	2.32995

B 6 18
B 6 3

Eps=11.50
EpsInf=2.0449
HbondAcidity=0.229
HbondBasicity=0.265
SurfaceTensionAtInterface=61.24
CarbonAromaticity=0.2142
ElectronegativeHalogenicity=0

#P b3lyp/tzvp opt em=gd3bj freq nosymm scrf=(SMD,Solvent=Generic,Read)

intermediate

-2 1

N	1.46514	-0.54422	0.11168
C	2.56633	-0.23490	-0.01905
C	3.96127	0.13361	-0.18706
C	4.11526	1.16206	-1.19974
N	4.25492	1.97105	-2.00673
H	4.43639	0.61490	1.85609
O	4.55218	-2.16182	0.20199
C	4.89347	-1.16528	-0.45823
O	5.83379	-0.98541	-1.23754
H	4.60140	-1.90226	2.24172
N	4.65930	-3.34769	5.02895
C	4.79959	-2.53859	4.22217
C	4.95448	-1.51004	3.20973
C	6.34966	-1.14222	3.04228
N	7.45105	-0.83339	2.91200
O	3.08242	-0.39008	4.26003
C	4.02286	-0.21073	3.48073
O	4.36462	0.78560	2.82043

Eps=11.50
EpsInf=2.0449
HbondAcidity=0.229
HbondBasicity=0.265
SurfaceTensionAtInterface=61.24
CarbonAromaticity=0.2142
ElectronegativeHalogenicity=0

#P b3lyp/tzvp opt=(ts,calcfc,noeigen,ModRedundant) em=gd3bj freq nosymm scrf=(SMD,Solvent=Generic,Read)

TS2

-2 1

N	1.65898600	-0.52896300	0.80606900
C	2.67290200	-0.35606700	0.26055400
C	3.90709700	-0.16239900	-0.36837200
C	4.07665200	0.97135100	-1.17013100
N	4.23098200	1.92197700	-1.82384100
H	4.59720000	0.74041700	1.77744200
O	4.73789600	-2.19672600	0.48782100
C	4.92491600	-1.23565800	-0.36605700
O	5.89326600	-1.17787100	-1.14627400
H	4.68945200	-1.73380000	1.82223800
N	4.44137700	-3.48571500	4.43836300
C	4.65155100	-2.53642500	3.82155100
C	4.90087300	-1.37698600	2.99651500
C	6.29864500	-1.00183400	2.99142200
N	7.40789400	-0.69352500	2.94713800
O	3.15873400	-0.26241700	4.22865000
C	3.97902300	-0.21136500	3.34960100
O	4.14917400	0.90377400	2.63138100

B 10 13
B 10 7

Eps=11.50
EpsInf=2.0449
HbondAcidity=0.229
HbondBasicity=0.265
SurfaceTensionAtInterface=61.24
CarbonAromaticity=0.2142
ElectronegativeHalogenicity=0

#P b3lyp/tzvp opt em=gd3bj freq nosymm scrf=(SMD,Solvent=Generic,Read)

[E-C(CN)$_2$COOH]$_2^{2-}$

-2 1

N	1.52711	-0.42534	-0.01712
C	2.65959	-0.21127	-0.02514
C	4.07458	0.10302	-0.12341
C	4.24043	1.14892	-1.11617
N	4.29720	1.96606	-1.92702
H	4.55988	0.61179	1.93407
O	4.57352	-2.22381	0.18010
C	4.97160	-1.21669	-0.42624
O	5.93550	-1.02807	-1.17161
H	4.28072	-2.20557	1.16265
N	5.02533	-3.27058	5.05594
C	4.88330	-2.48993	4.22025
C	4.80273	-1.49099	3.16963
C	6.15489	-1.11952	2.78799
N	7.25020	-0.85958	2.54149
O	3.07865	-0.40767	4.46570
C	3.90033	-0.20340	3.56986
O	4.14244	0.79675	2.87523

Eps=11.50
EpsInf=2.0449
HbondAcidity=0.229
HbondBasicity=0.265
SurfaceTensionAtInterface=61.24
CarbonAromaticity=0.2142
ElectronegativeHalogenicity=0

#P b3lyp/tzvp opt em=gd3bj freq nosymm scrf=(SMD,Solvent=Generic,Read)

[Z-C(CN)$_2$COOH]$_2^{2-}$

-2 1

N	-1.49591	3.79537	5.32872
C	-0.50231	4.04694	4.78064
C	0.70057	4.30982	4.11542
C	0.93019	5.59900	3.61730
N	1.12186	6.67081	3.21386
H	3.61920	2.08090	2.95919
O	1.49385	2.07612	4.48448
C	1.69814	3.29262	3.89636
O	2.70263	3.43504	3.21596
H	2.12640	1.34652	4.57715
N	4.45947	-3.36451	4.22525
C	4.65041	-2.29277	3.82129
C	4.87912	-1.00367	3.32256
C	6.08188	-0.74009	2.65741
N	7.07530	-0.48806	2.10920
O	2.87648	-0.13027	4.22191
C	3.88105	0.01297	3.54183
O	4.08493	1.22996	2.95434

Eps=11.50
EpsInf=2.0449
HbondAcidity=0.229
HbondBasicity=0.265
SurfaceTensionAtInterface=61.24
CarbonAromaticity=0.2142
ElectronegativeHalogenicity=0
